# Mechanisms by which autophagy regulates memory capacity in ageing

**DOI:** 10.1111/acel.13189

**Published:** 2020-07-30

**Authors:** Maria De Risi, Giulia Torromino, Michele Tufano, Stéphanie Moriceau, Annabella Pignataro, Manon Rivagorda, Nicolò Carrano, Silvia Middei, Carmine Settembre, Martine Ammassari‐Teule, Fabrizio Gardoni, Andrea Mele, Franck Oury, Elvira De Leonibus

**Affiliations:** ^1^ Telethon Institute of Genetics and Medicine Telethon Foundation Pozzuoli Italy; ^2^ Institute of Biochemistry and Cell Biology (IBBC) National Research Council Rome Italy; ^3^ Institut National de la Santé et de la Recherche Médicale (INSERM) U1151 Institut Necker Enfants‐Malades (INEM) Université Paris Descartes‐Sorbonne–Paris Cité Paris France; ^4^ Laboratory of Psychobiology Department of Experimental Neurology Santa Lucia Foundation Rome Italy; ^5^ Institute of Translational Pharmacology (IFT) National Research Council Rome Italy; ^6^ Department of Pharmacological and Biomolecular Sciences University of Milan Milan Italy; ^7^ Department of Biology and Biotechnology "C. Darwin" Sapienza University of Rome Rome Italy; ^8^ Center for Research in Neurobiology "D. Bovet" Sapienza University of Rome Rome Italy

**Keywords:** ageing, amyloid fibrils, alpha‐synuclein, autophagy, GluA1, mild cognitive impairment, Spermidine

## Abstract

Autophagy agonists have been proposed to slow down neurodegeneration. Spermidine, a polyamine that acts as an autophagy agonist, is currently under clinical trial for the treatment of age‐related memory decline. How Spermidine and other autophagy agonists regulate memory and synaptic plasticity is under investigation. We set up a novel mouse model of mild cognitive impairment (MCI), in which middle‐aged (12‐month‐old) mice exhibit impaired memory capacity, lysosomes engulfed with amyloid fibrils (β‐amyloid and α‐synuclein) and impaired task‐induced GluA1 hippocampal post‐translation modifications. Subchronic treatment with Spermidine as well as the autophagy agonist TAT‐Beclin 1 rescued memory capacity and GluA1 post‐translational modifications by favouring the autophagy/lysosomal‐mediated degradation of amyloid fibrils. These findings provide new mechanistic evidence on the therapeutic relevance of autophagy enhancers which, by improving the degradation of misfolded proteins, slow down age‐related memory decline.

## Graphical abstract

We developed a novel mouse model of mild cognitive impairment (MCI) that allows to identify middle‐aged (12‐month‐old) mice with impaired memory capacity and vulnerable to age‐dependent memory decline. Spermidine and TAT‐Beclin 1, by stimulating autophagy/lysosomal degradation of misfolded protein, reduce the amyloid load and rescue the memory‐dependent post‐translational modifications of GluA1 receptors in the hippocampus of the cognitively impaired ageing mice.

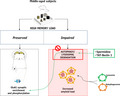

## 1 INTRODUCTION

Mild cognitive impairment (MCI) is an intermediate condition between healthy ageing and dementia, characterized by deficits in at least one cognitive domain without major repercussions in daily life and it is identifiable using sophisticated neuropsychological tests already in middle‐aged subjects (Petersen et al., 1999). MCI has been associated with impaired synaptic connectivity due to reduced glutamate transmission (Zeydan et al., 2017). However, the mechanisms responsible for MCI are still unknown.

Evidence in humans suggests that MCI deficits pinpoint increased β‐amyloid (Aβ) load (Hansson et al., 2006), which is one of the major pathological marker of Alzheimer's disease (AD). Experimental models have consistently reported that age‐dependent accumulation of misfolded proteins, such as Aβ and α‐synuclein (α‐syn), is sufficient to impair glutamate receptors trafficking (Guntupalli, Widagdo, & Anggono, 2016). Indeed, increased Aβ levels cause a reduction of synaptic density and an induction of synaptic depression in pyramidal neurons by modulating the localization of glutamate receptor 1 subunit (GluA1) of the α‐amino‐3‐hydroxy‐5‐methyl‐4‐isoxazolepropionic acid (AMPA) receptors (hereon called GluA1 receptors) at the synapses (Hsieh et al., 2006) and by inhibiting hippocampal long‐term potentiation (Walsh et al., 2005).

MCI occurs in only some middle‐aged individuals, while others are spared throughout their entire life. The identification of MCI with prognostic value for developing dementia is crucial for identifying the disease mechanisms preceding neurodegeneration and for starting restorative or preventive therapies before its onset.

One of the most promising pharmacological strategies that is currently being tested is the use of autophagy agonists. Autophagy is a cellular catabolic process whereby proteins and organelles are transported to lysosomes for degradation, by means of autophagosomes, which are double‐membrane intracellular vesicles. Induction of autophagy is crucial for the degradation of aggregated proteins, such as Aβ, tau or α‐syn, which are known to disrupt cognitive functions during ageing (for review see Yue, Friedman, Komatsu, & Tanaka, 2009). More recent experimental evidence suggests that autophagy might be also involved in functional mechanisms for hippocampal‐dependent memory formation and maintenance of cognitive fitness throughout life (Glatigny et al., 2019; Hylin et al., 2018).

Major problems of using these drugs in the clinic are that the mechanisms by which they influence memory are not known and that they might have dramatic side effects. However, a recent clinical trial is testing long‐term treatment with Spermidine (a “longevity treatment”) (Eisenberg et al., 2016; Tain et al., 2020), a polyamine that is naturally present in living organisms and is involved in the maintenance of physiological homeostasis (Madeo, Carmona‐Gutierrez, Kepp, & Kroemer, 2018). Data on *Drosophila melanogaster* reported that Spermidine ameliorates memory during ageing (Bhukel et al., 2019; Bhukel, Madeo, & Sigrist, 2017; Sigrist et al., 2014).

In a recent clinical trial in elderly subjects, Spermidine was shown to be effective in improving hippocampal‐dependent memory in a group of elderly people with MCI and thus at risk of AD development (Wirth et al., 2018).

Spermidine has been known for many years for its role as a polyamine that modulates the activity of N‐methyl‐D‐aspartate (NMDA)‐type and Ca^2+^ permeable AMPA glutamate receptors (Williams, Romano, Dichter, & Molinoff, 1991). Although its effects on memory in ageing have been also linked to its action as an autophagy enhancer (Eisenberg et al., 2009; Gupta et al., 2013; Pietrocola et al., 2015), how Spermidine restores memory by stimulating autophagy is still under investigation.

In this study, we first established an animal model of “pathological ageing.” We identified memory‐impaired middle‐aged (12‐month‐old) mice with a task that requires a high memory load capacity (the 6 different objects task, 6‐DOT) that we have previously showed to rely on phosphorylation of GluA1 receptors at serines 845 (S845) and 831 (S831) in the hippocampus (Olivito et al., 2016). Middle‐aged mice with impaired memory capacity (MC), as compared to age‐matched memory preserved subjects, further decline in memory performance with ageing (at 18 months of age) and present impaired hippocampal GluA1 phosphorylation, associated with increased misfolded proteins and defective autophagy/lysosomal degradative capacity. Subchronic treatment with Spermidine rescues the memory‐dependent post‐translational modifications of GluA1 receptors by favouring misfolded protein lysosomal degradation.

## 2 RESULTS

### 2.1 Reduced memory capacity in middle‐aged subjects predicts age‐dependent memory decline

**Figure 1:**
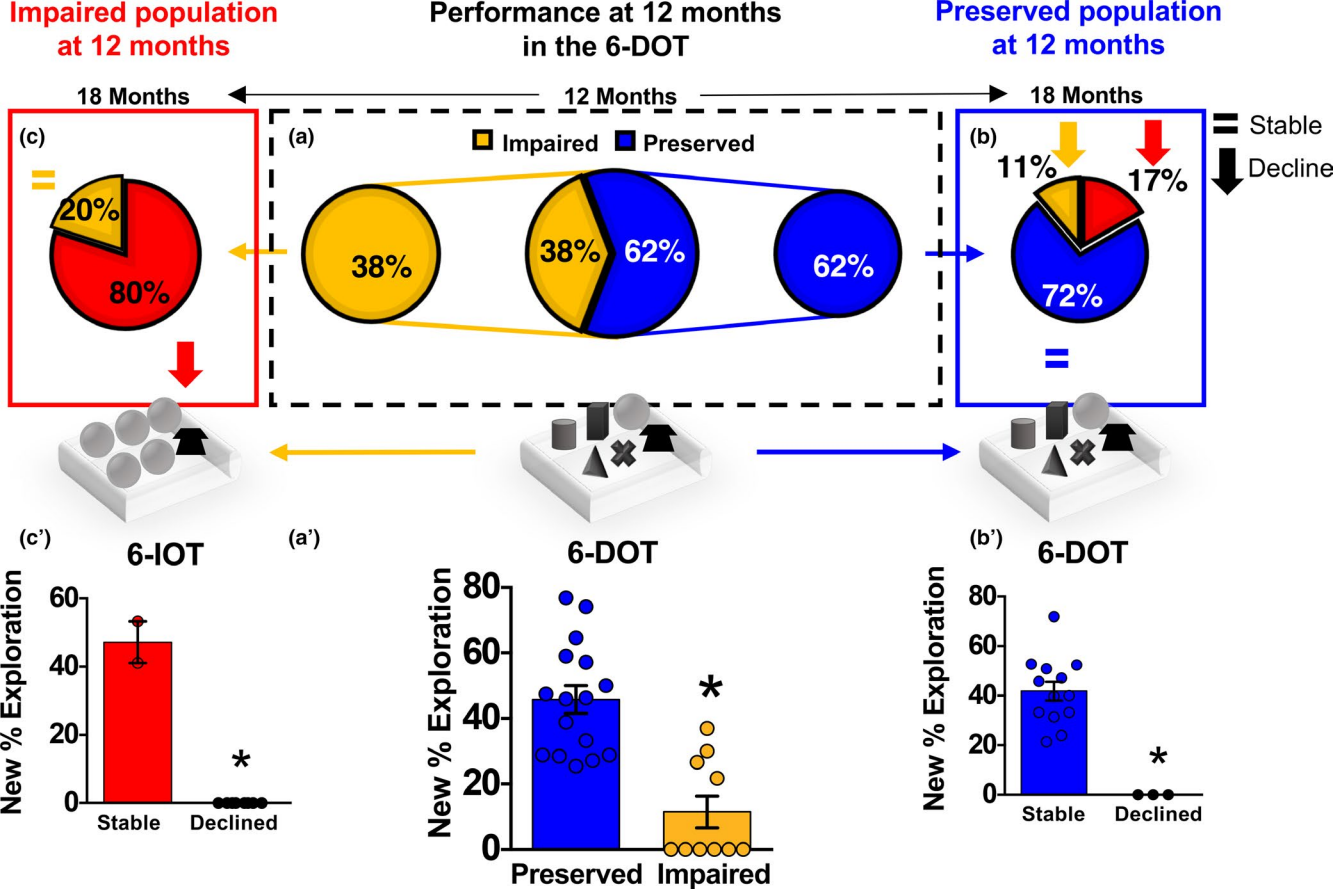
Inter‐individual variability in memory capacity in a middle‐aged population of outbred mice predicts age‐related memory decline. (a) A population of 12‐month CD1 outbred mice was tested in the 6‐DOT (high memory load task). 62% showed preserved MC (*Preserved*), while 38% exhibited bad performance (*Impaired*) [*χ* ^2^ = 55.1200; *df* = 1; *p* < 0.0001] [*Preserved* *n* = 16; *Impaired* *n* = 10]. Both subgroups were then tested at 18 months. (b) The *Preserved* group was tested in the 6‐DOT (blue panel on the right). Among the aged *Preserved* mice, we found that the majority (72% ‐ *Stable*; blue) was spared from ageing‐related MC decline, while the remaining 28% (*Declined*) was impaired [*χ* ^2^ = 72.5925; *df* = 1; *p* < 0.0001] [*Stable* *n* = 13; *Declined* *n* = 3]. This latter subgroup was then tested in the 6‐IOT, where 11% (yellow) showed good performance while 17% (red) was found impaired also in the 6‐IOT. (c) The *Impaired* group was directly tested in the 6‐IOT at 18 months (red panel on the left). We found that 80% (red) further declined to bad performance in the 6‐IOT, while 20% (yellow) was found stable [*Stable* *n* = 2; *Declined* *n* = 8]. (a') Performance of the subjects at 12 months in the 6‐DOT [one‐way ANOVA *F*_1, 24_ = 27.088; *p* < 0.0001]. (b') Performance of mice from the *Preserved* subgroup in the 6‐DOT at 18 months [one‐way ANOVA *F*_1, 14_ = 26.394; *p* < 0.0001]. (c') The percentage exploration of the new object in the 6‐IOT at 18 months of mice from the *Impaired* subgroup at 12 months [one‐way ANOVA *F*_1, 8_ = 386.804; *p* < 0.0001]. Bar charts represent mean ± *SEM*. *p < 0.05 between groups.

Reduced memory capacity, the number of elements that a subject can remember in a short‐time interval, is a clinically relevant symptom in MCI and prodromal to AD (Belleville, Chertkow, & Gauthier, 2007). We hypothesized that challenging the brain with high memory load could pinpoint subtle deficits in cognitive processing in middle‐aged subjects, which would be undetectable using a low load task. To test this idea in rodents, an outbred population of middle‐aged (12‐month‐old) mice (CD1) was exposed to the 6‐DOT, which is the highest number of objects that young mice can memorize (Sannino et al., 2012). Based on individual performance (see methods) in the discrimination of the new object (New % Exploration) (Figure 1a'), 62% of the population was included in the subgroup of *Preserved*, while the remaining 38% was classified as *Impaired* (Figure 1a). *Preserved* and *Impaired* groups showed similar performance in the 6‐identical objects task (6‐IOT), which requires low memory capacity as all 6 objects are identical (Figure S1a), in line with the current literature showing middle‐aged mice are not impaired in the classical version of the novel object recognition task, made with only 2 identical objects (Bergado, Almaguer, Rojas, Capdevila, & Frey, 2011). Thus, the memory impairment that we found in the 38% of the population was specific for the high memory load conditions, and this was not due to reduced propensity to explore the objects during the study phase (Table S1).

To test the hypothesis that inter‐individual MC differences at 12 months could predict MC decline in elderly subjects at 18 months, as observed for corresponding stages in humans (Zanchi et al., 2017), the same mice were re‐tested at 18 months (longitudinal study): 80% of the subjects that were impaired in the 6‐DOT at 12 months declined in performance also in the 6‐IOT (defined as *Declined*) (Figure 1c left panel in red and Figure 1c'). In contrast, mice belonging to the *Preserved* group, which were not impaired in the 6‐DOT at 12 months (Figure 1a, right panel), maintained a good performance at 18 months (defined as *Stable*) [72% (*Stable*—blue in Figure 1b) *vs* 28% (*Declined*—red and orange in Figure 1b)] both in the 6‐DOT (Figure 1b') and in the 6‐IOT (Figure S1b). Thus, performance in the 6‐DOT at 12 months identified subjects that were vulnerable (*Impaired*) or resistant (*Preserved*) to age‐dependent memory decline.

### 2.2 Impaired memory capacity in middle‐aged subjects is associated with defective degradation of misfolded proteins, which is rescued by Spermidine

To underpin the molecular mechanisms that determine middle‐aged *Preserved* and *Impaired* mice, a new group of 12‐month‐old mice was challenged in the 6‐DOT to identify *Preserved* (pMC) and *Impaired* (iMC) subjects each compared to a young exploration‐matched companion (young‐paired, YP; see Table S2 for objects exploration during the study phase). *Preserved* mice had a performance similar to YP, while *Impaired* showed a strong reduction in the exploration of the novel object, suggesting an impaired MC (Figure S2a). MCI in humans occurs at an early stage, preceding neurodegeneration (Lleo et al., 2019), and, indeed, *Impaired* mice did not show signs of neuronal loss in any of the hippocampal subfields (CA1, CA3 and dentate gyrus—DG) or impaired levels of pre‐ and postsynaptic markers, such as synaptophysin and postsynaptic density protein 95 (PSD95) (Figure S2b–g).

**Figure 2:**
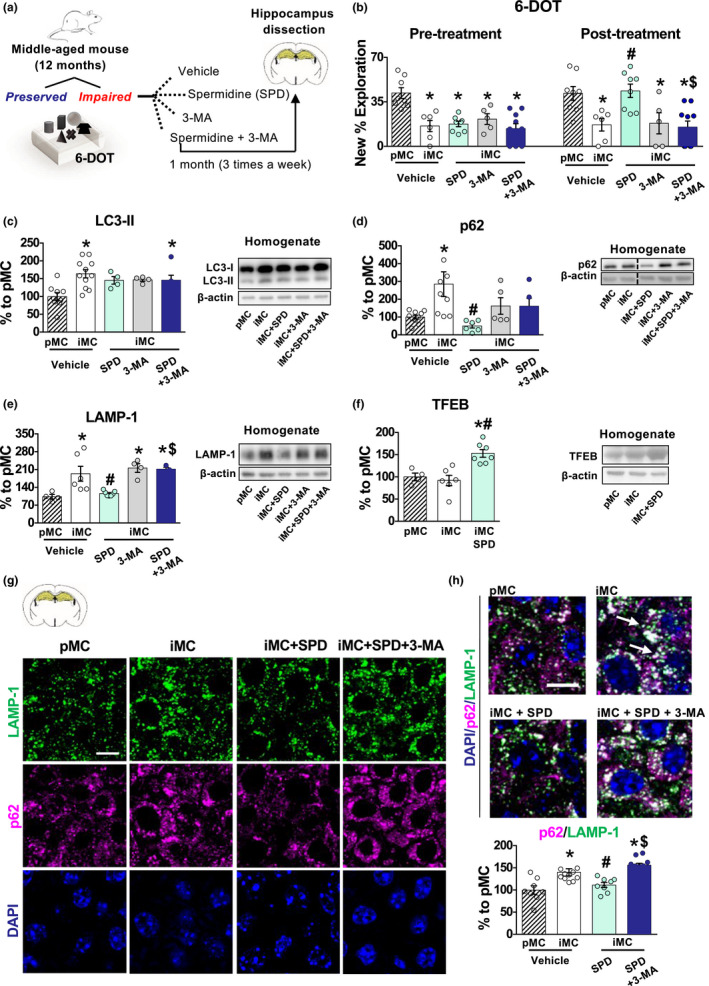
Middle‐aged *Impaired* subjects show impaired autophagy which is rescued by Spermidine subchronic treatment. (a) Schematic representation of the experimental procedure: 12‐month‐old mice were tested in the 6‐DOT and divided into *Preserved* (pMC) and *Impaired* (iMC). The pMC group was treated with vehicle, while iMC subjects were additionally subdivided into four groups and subchronically treated with vehicle, Spermidine, 3‐MA or Spermidine+ 3‐MA. After 1 month of 3 intraperitoneal injections *per* week, mice again underwent the 6‐DOT. Hippocampi were dissected immediately at the end of the procedure, and protein extraction was performed on homogenates and synaptosome fractions. (b) iMC vehicle‐treated mice showed impaired performance as compared to pMC [*F*_4, 32_ = 9.74, *p* < 0.0001], as evidenced by the percentage of exploration of the new object [pMC *n* = 8; iMC=6; iMC SPD *n* = 8; iMC 3‐MA *n* = 5; iMC SPD+ 3‐MA *n* = 10], before treatment all groups explored the new object significantly less than the pMC group. Subchronic treatment with Spermidine rescued performance in the iMC mice [pretreatment: *F*_1, 25_ = 5.49, *p* < 0.0273; treatment: *F*_1, 25_ = 4.19; *p* = 0.0513; pretreatment × treatment: *F*_1, 25_ = 6.48, *p* = 0.0175], an effect that was prevented by pretreatment with 3‐MA. (c–e) Western blot analysis on iMC vehicle treated as compared to pMC vehicle showed a significant increase of (c) LC3‐II [*F*_4, 29_ = 4.96, *p* = 0.0036], (d) p62 [*F*_4, 29_ = 4.81, *p* = 0.0042] and (e) LAMP‐1 [*F*_4, 19_ = 8.17, *p* = 0.0005]. Treatment with Spermidine (c) did not change the level of LC3‐II but (d) significantly decreased p62 [treatment: *F*_1, 20_ = 4.91, *p* = 0.0384; pretreatment x treatment: *F*_1, 20_ = 4.82, *p* = 0.0400] and (e) LAMP‐1 [pretreatment: *F*_1, 16_ = 8.20, *p* = 0.0112] in vehicle‐treated iMC subjects. All these effects were blocked by 3‐MA administration (c–e). Representative bands for each condition are reported [pMC vehicle *n* ≥ 4; iMC vehicle *n* ≥ 5; iMC SPD *n* ≥ 4; iMC 3‐MA *n* ≥ 3; iMC SPD+ 3‐MA *n* ≥ 4]. (f) TFEB expression was not different between pMC and iMC; however, Spermidine treatment significantly increased its expression in iMC mice [*F*_2, 14_ = 11.99, *p* = 0.0009]. Representative bands for each condition are presented [pMC *n* = 4; iMC vehicle *n* = 6; iMC SPD *n* = 7]. (g, h) LAMP‐1/p62 colocalization analysis [*F*_3, 34_ = 13.22, *p* < 0.0001] showed that iMC group has increased colocalization between p62 and LAMP‐1 in CA3 hippocampal subfield as compared to pMC, which was reduced by Spermidine treatment and reinstated by 3‐MA pretreatment. Representative images of p62 and LAMP‐1 immunofluorescence are reported in (g) and representative images of LAMP‐1/p62 colocalization are reported in (h). White arrows indicate LAMP‐1/p62 colocalization [pMC vehicle *n* = 8; iMC vehicle *n* = 11; iMC SPD *n* = 8; iMC SPD+ 3‐MA *n* = 11]. Bar charts represent mean ± *SEM*. Scale bar: 10 µm. *p < 0.05 vs. pMC; #*p* < 0.05 vs. iMC vehicle; $*p* < 0.05 vs. iMC SPD. [SPD = Spermidine].

We used this model of MCI to test the effects of Spermidine (intraperitoneally injected for three times per week for 1 month) in *Impaired* middle‐age mice. To this aim, we treated *Impaired* mice with: vehicle, Spermidine, the autophagy inhibitor 3‐MA (Blommaart, Krause, Schellens, Vreeling‐Sindelarova, & Meijer, 1997) or a combination of Spermidine and 3‐MA (Figure 2a), to assess the contribution of autophagy in mediating the behavioural effects of Spermidine. The doses of Spermidine (50 mg/kg) and 3‐MA were chosen based on previous studies in the literature and on pilot experiments in young mice showing that they efficiently activate and inhibit autophagy, respectively (Figure S3a–b).

We found that 1 month of subchronic treatment with Spermidine rescued the memory impairment of *Impaired* mice, but this effect was prevented by the contemporary administration of 3‐MA, suggesting that the rescue effect of Spermidine was autophagy‐dependent (Figure 2b). Of note, this observation was not a result of nonspecific effects of Spermidine on the exploration or locomotor activity (Tables S3‐S4). Thus, we tested the state of autophagy in the hippocampus of *Impaired* mice compared to *Preserved* ones and the effect of Spermidine on it. Western blot analysis showed that *Impaired* mice had significantly increased accumulation of the microtubule‐associated protein 1A/1B‐light chain 3 (LC3‐II), a protein of the autophagosomal membrane, of the sequestosome 1 (p62/SQSTM1), which is a specific autophagy substrate (Mizushima, Yoshimori, & Levine, 2010), and of the lysosomal‐associated membrane protein 1 (LAMP‐1), a lysosomal marker, whose increased expression is associated with lysosomes accumulation (Rami, Benz, Niquet, & Langhagen, 2016) (Figure 2c–e). The increase of LC3‐II, p62 and LAMP‐1 in *Impaired* mice suggests an impairment in the late stages of the autophagic flux. Indeed, increased p62/LAMP‐1 colocalization in *Impaired* mice, detected by immunofluorescence, suggests that the autophagosomal/lysosomal fusion occurs, but lysosomal degradation is impaired (Figure 2g–h). Treatment with Spermidine restored the autophagic cargo degradation as demonstrated by a decrease of p62 and LAMP‐1 levels (Figure 2d–e) and reduced colocalization between p62 and LAMP‐1 puncta (Figure 2g–h). Chronic treatment with Spermidine by favouring the degradation of misfolded proteins reduced the degradative need in *Impaired* mice, which resulted in a net normalization of LAMP1 after 1 month of treatment. Spermidine was recently shown to promote the synthesis, hence the activation, of the lysosomal/autophagy transcription factor EB (TFEB), which in turn promotes lysosome/autophagy pathway in old human B cells (Zhang et al., 2019). Consistent with this, we found that Spermidine treatment increased TFEB levels in *Impaired* mice (Figure 2f) suggesting a potential mechanism for the effect of Spermidine in treated mice. Autophagy markers were not changed in the 3‐MA group compared to vehicle‐*Impaired* mice, but vehicle‐*Impaired* mice had per se a high level of p62, LC3‐II and LAMP‐1 (Figure 2c–e); thus, an additional effect of 3‐MA was likely not evident due to a ceiling effect. However, its administration was sufficient to prevent Spermidine‐induced rescue of lysosomal cargo degradation (Figure 2d–e) in line with the observation that phosphatidylinositol 3‐phosphate (PI3P) production, which is inhibited by 3‐MA (Blommaart et al., 1997), is required not only for autophagosome biogenesis but also for proper lysosome biogenesis and function (Bartolomeo et al., 2017).

**Figure 3:**
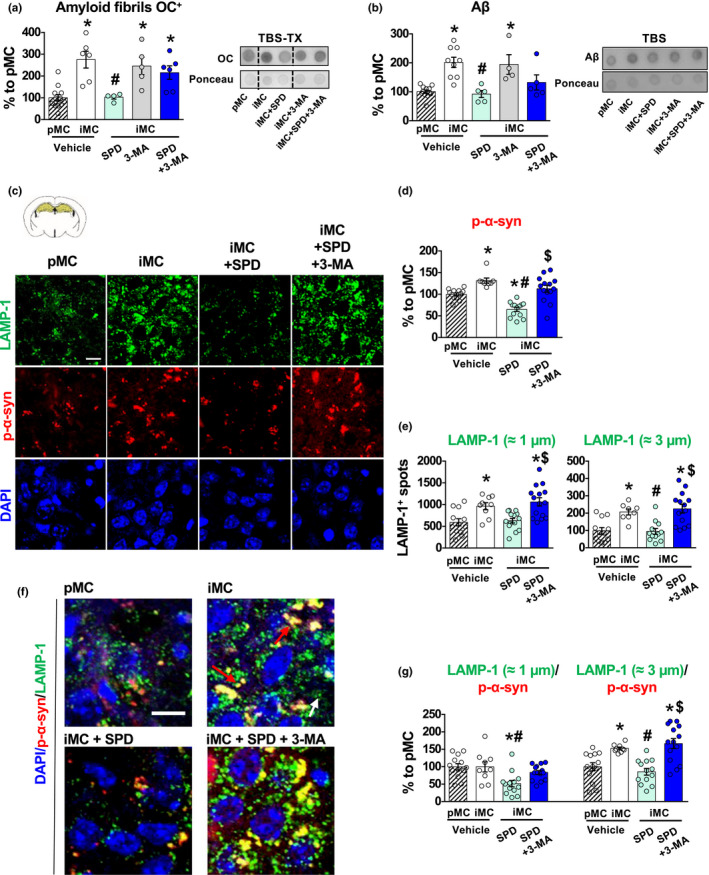
Middle‐aged *Impaired* subjects show increased amyloid load which is reduced by Spermidine. (a, b) iMC vehicle‐treated mice showed a significant increase of OC^+^ amyloid fibrils in the TBS‐TX extracts [*F*_4, 28_ = 9.61, *p* < 0.0001] and of Aβ in the TBS extracts [*F*_4, 30_ = 10.09, *p* < 0.0001], compared to pMC vehicle‐treated mice. Spermidine treatment reduced both OC^+^ amyloid fibrils in the TBS‐TX fraction and Aβ in the TBS fraction. These effects were prevented by 3‐MA administration [OC^+^ amyloid fibrils: treatment: *F*_1, 17_ = 8.50, *p* = 0.0096; pretreatment × treatment: *F*_1, 17_ = 4.18, *p* = 0.0566; Aβ: treatment: *F*_1, 19_ = 13.90, *p* = 0.0014]. Representative dot blots for each condition are presented [pMC *n* ≥ 5; iMC vehicle *n* ≥ 4; iMC Spermidine *n* = 4; iMC 3‐MA *n* ≥ 4; iMC SPD+ 3‐MA *n* = 6]. (c–e) Representative images of LAMP‐1 and p‐α‐syn immunofluorescence (c) and relative quantification (d, e). iMC show increased accumulation of p‐α‐syn compared to pMC in CA3 hippocampal subfield. Spermidine reduced this accumulation if not combined with 3‐MA pretreatment [*F*_3, 44_ = 20.48, *p* < 0.0001]. (e) LAMP‐1^+^ spots of ≈1 µm diameter (small) and spots of ≈3 µm (large) were evaluated. LAMP‐1 quantification showed that vehicle‐treated iMC has an increased number of LAMP‐1^+^ spots independently from the diameter, compared to pMC. Spermidine reduced both types of LAMP‐1^+^ spots but this effect was blocked by 3‐MA [small spots: *F*_3, 46_ = 8.88, *p* < 0.0001; large spots: *F*_3, 42_ = 11.25, *p* < 0.0001]. (f) Colocalization of p‐α‐syn with LAMP‐1 was increased only in large spots of iMC vehicle‐treated mice, compared to pMC. Again, Spermidine reduced this colocalization in both types of LAMP‐1 spots, but not when in combination with 3‐MA [small spots: *F*_3, 45_ = 6.05, *p* = 0.0015; large spots: *F*_3, 45_ = 12.03, *p* < 0.0001] [pMC *n* = 13; iMC vehicle *n* = 9; iMC Spermidine *n* = 14; iMC SPD+ 3‐MA *n* = 15]. White arrows indicate LAMP‐1 small spot, while red arrows indicate LAMP‐1 large spots colocalizing with p‐α‐syn. Bar charts represent mean ± *SEM*. Scale bar: 10 µm. *p < 0.05 vs. pMC; #*p* < 0.05 vs. iMC vehicle; $*p* < 0.05 vs. iMC SPD. [SPD = Spermidine].

Increased LAMP‐1 in *Impaired* mice is associated with the presence of large lysosomes engulfed with aggregated proteins. Impaired autophagy/lysosomal protein degradation has been associated with the accumulation of misfolded proteins. Therefore, we performed a dot blot analysis to evaluate the presence of amyloid fibrils, using the OC antibody (OC^+^) in Tris‐buffered saline (TBS) containing 1% TX‐100 (TBS‐TX) and TBS fractions, which are generally used to evaluate intracellular and extracellular protein aggregate load, respectively (Pignataro et al., 2019). The OC antibody recognizes epitopes common to different types of amyloid fibrils, including those made of Aβ and α‐syn aggregates (Kayed et al., 2007), hereon defined as “amyloid load.” *Impaired* mice showed a significant TBS‐TX accumulation of OC^+^ amyloid fibrils compared to *Preserved* mice (Figure 3a). Concomitantly, *Impaired* mice showed also increased TBS Aβ (Figure 3b), detected with an antibody that recognizes the monomeric form of Aβ, specific for C‐terminals amino acids 37‐42. Both OC^+^ amyloid fibrils in TBS‐TX and Aβ in TBS extracts were decreased by Spermidine in *Impaired* mice (Figure 3a,b). None of the treatment affected OC^+^ amyloid fibrils in the TBS fraction and Aβ in TBS‐TX fraction with Spermidine or 3‐MA treatment, in line with a lack of differences between *Preserved* and *Impaired* vehicle‐treated subjects (Figure S4a‐b).

To understand if the effects was extended also to α‐syn and to define the cellular localization of these aggregated proteins, we conducted an immunofluorescence analysis against the phosphorylation residue S129 of α‐syn (p‐α‐syn) that is a marker of α‐syn protein aggregates (oligomers and fibrils) (Fujiwara et al., 2002). *Impaired* mice showed an increased level of p‐α‐syn compared to *Preserved* ones (Figure 3c,d), which was decreased by Spermidine treatment.

Misfolded proteins are degraded by the autophagosomal/lysosomal system that we have shown being defective in *Impaired* mice. We hypothesized that the increased amyloid load in *Impaired* mice accumulated into lysosomes, similarly to what happens in genetic animal models of autophagy impairment (Monaco et al., 2020; Nixon et al., 2005; D. S. Yang et al., 2011). Thus, we analysed in more depth the effects of Spermidine on lysosomes and p‐α‐syn.

The normal diameter of lysosomes generally does not exceed 1 µm (Bandyopadhyay, Cyphersmith, Zapata, Kim, & Payne, 2014). We conducted a morphometric analysis of LAMP‐1 by immunofluorescence, binning LAMP‐1^+^ spots into two categories, small and large, which were defined as those having a diameter of about 1 or 3 µm, respectively (Figure 3e). At the same time, we measured their colocalization with p‐α‐syn. *Impaired* mice had a higher number of both small and large lysosomes as compared to *Preserved* ones, but we found a significant increase in the colocalization between p‐α‐syn and LAMP‐1 exclusively in the large subtype (Figure 3f,g), in accordance with the fact that large lysosomes are associated with impaired lysosomal function (Bandyopadhyay et al., 2014). Interestingly, Spermidine reduced p‐α‐syn/LAMP‐1 colocalization to *Preserved* mice levels (Figure 3f,g). The observation that most, if not all, of the effects of Spermidine were blocked by 3‐MA (Figure 3) suggests that Spermidine reduces the amyloid load by increasing the degradative capacity of lysosomes.

### 2.3 Spermidine rescues memory capacity impairment re‐establishing GluA1 synaptic functions in middle‐aged subjects

According to previous findings showing that performance in the 6‐DOT requires the phosphorylation of the GluA1 subunit of AMPA receptors at S845 and S831 in the hippocampus (Olivito et al., 2016), in young animals exposure to the 6‐DOT induced GluA1 synaptic enrichment in the synaptosome (Figure S5a‐b), accompanied by increased phosphorylation of both S845 (Figure S5a‐b) and S831 (Figure S5a‐b) at the synaptic level. This evidence is in line with previous findings in young animals showing that mutations of these two serine sites are sufficient to impair MC (Olivito et al., 2016). Therefore, we tested the hypothesis that *Impaired* middle‐aged subjects might fail to activate this process in response to the task and whether Spermidine also rescues the synaptic mechanisms involved in this process.

**Figure 4:**
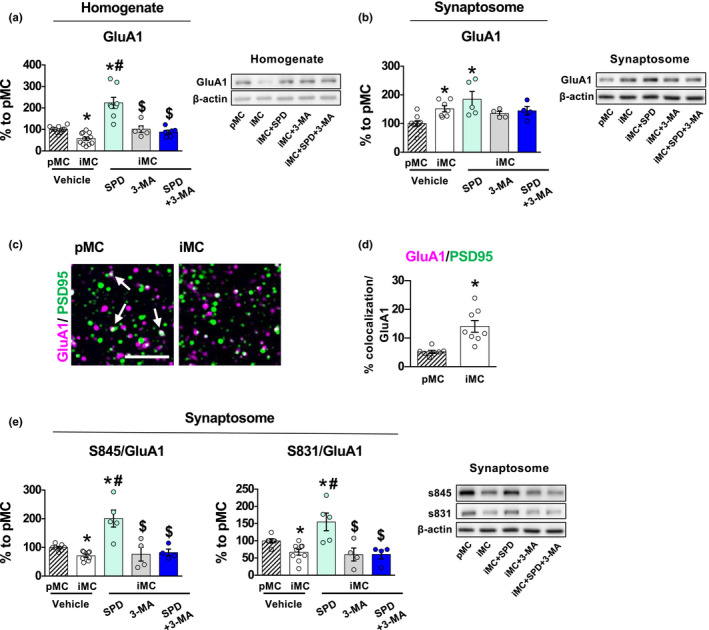
Subchronic treatment with Spermidine rescues the memory capacity task‐induced GluA1 post‐translational modifications *via* autophagy. (a, b) GluA1 subunit expression was found significantly reduced in the homogenate fraction of vehicle‐treated iMC mice compared to vehicle‐treated pMC [*F*_4, 33_ = 22.3, *p* < 0.0001] (a) but it was increased in the synaptosomal fraction [*F*_4, 24_ = 5.24, *p* = 0.0035] (b). Spermidine treatment increased GluA1 in the homogenate fraction, an effect that was prevented by 3‐MA pretreatment [pretreatment: *F*_1, 27_ = 7.88, *p* = 0.0091; treatment: *F*_1, 27_ = 21.90, *p* < 0.0001; pretreatment × treatment: *F*_1, 27_ = 30.23, *p* < 0.0001]. Representative bands for each condition are reported on the right [pMC n ≥ 4; iMC vehicle *n* ≥ 5; iMC Spermidine *n* ≥ 5; iMC 3‐MA *n* ≥ 4; iMC SPD+ 3‐MA *n* ≥ 4]. (c, d) The % of colocalized GluA1^+^ and PSD95^+^ spots was increased in iMC relative to the total level of GluA1 compared to pMC [*F*_1, 13_ = 8.51, *p* = 0.0120] in CA3 hippocampal subfield. Representative images of GluA1/PSD95 colocalization (white arrows) are reported in (c). Scale bar: 5 µm. (e) The synaptic enrichment of GluA1 in iMC was accompanied by a significant reduction of the phosphorylation of S845 and S831 [S845: *F*_4, 23_ = 12.11, *p* < 0.0001; S831: *F*_4, 25_ = 8.69, *p* = 0.0001], which was rescued by Spermidine treatment [S845: pretreatment: *F*_1, 17_ = 9.25, *p* = 0.0074; treatment: *F*_1, 17_ = 13.56, *p* = 0.0018; pretreatment × treatment: *F*_1, 17_ = 11.34, *p* = 0.0037; S831: pretreatment: *F*_1, 17_ = 8.36, *p* = 0.0101; treatment: *F*_1, 17_ = 7.58, *p* = 0.0136; pretreatment × treatment: *F*_1, 17_ = 7.54, *p* = 0.0138]. All these effects were prevented by 3‐MA treatment. Representative bands for each condition are reported [pMC *n* = 4; iMC vehicle *n* = 5; iMC Spermidine *n* = 5; iMC 3‐MA *n* = 4; iMC SPD+ 3‐MA *n* = 4]. Representative bands are reported and belong to the same blot of GluA1 and β‐actin reported in (b). Bar charts represent mean ± *SEM*. *p < 0.05 vs. pMC; #*p* < 0.05 vs. iMC vehicle; $*p* < 0.05 vs. iMC SPD. [SPD = Spermidine].

Western blot analysis showed that *Impaired* mice had 43% reduction of GluA1 in the hippocampal homogenate (Figure 4a). Surprisingly, in the synaptosomal fraction, GluA1 of *Impaired* mice was significantly increased compared to *Preserved* mice (Figure 4b). To verify the localization of GluA1 at the synapses, we conducted an analysis of GluA1/PSD95 colocalization by immunofluorescence. We confirmed that the total amount of GluA1 was decreased in *Impaired* mice compared to *Preserved* ones, and it was associated with an equal expression of PSD95 (Figure S4c–e). The percentage of colocalization between GluA1/PSD95 spots, corrected for the total amount of GluA1, was higher in *Impaired* mice (Figure 4c,d), confirming the WB data showing a preferential localization of GluA1 at the synapses in this group. In parallel, we evaluated the level of S845 and S831 phosphorylation, as we know this is a required mechanism for high memory load performance (Olivito et al., 2016). Interestingly, we found that the increased GluA1 expression was accompanied by a decrease of S845 and S831 phosphorylation in *Impaired* mice compared to *Preserved* ones (Figure 4e). Thus, these results suggest that the synaptosomal accumulation of GluA1 in *Impaired* subjects is a compensatory nonfunctional mechanism activated when exposed to the high memory load in the 6‐DOT. This effect was specific for GluA1, as GluA2 and GluA3 subunit expression was not changed (Figure S4f–g), and it was not associated with changes at the transcriptional level, as evidenced by a lack of differences between *Preserved* and *Impaired* GluA1 mRNA levels (Figure S4h). Possible changes in the expression of protein kinase A (PKA), whose activation is responsible for S845 phosphorylation (Banke et al., 2000), could not account for the lower phosphorylation levels in *Impaired* mice (Figure S4I).

Remarkably, Spermidine treatment rescued the reduction of GluA1 expression in the homogenate (Figure 4a). Again, a significant increase of GluA1 was found in the synaptosomes after Spermidine treatment (Figure 4b). However, in contrast to vehicle‐*Impaired* mice, in this case GluA1 enrichment at the synapses was accompanied by an increase in S845 and S831 phosphorylation (Figure 4e), suggesting this time a functional activation of the GluA1 subunit at the synapses in response to the 6‐DOT request.

Spermidine has been shown to improve memory in young animals through PKA‐mediated phosphorylation of GluA1 in the hippocampus (Guerra et al., 2011). However, the dose we used in our study did not show this effect in young animals (Figure S3a^I^–a^II^). The rescue of GluA1 memory‐induced synaptic enrichment was abolished by the combination with 3‐MA (Figure 4a,b; Figure 4e), suggesting a direct involvement of autophagy in this process.

These findings provide the first evidence that Spermidine favours autophagy/lysosomal‐mediated amyloid load clearance in middle‐aged subjects. According to previous findings showing that soluble oligomeric Aβ induces dephosphorylation of GluA1 at S845 (Minano‐Molina et al., 2011), Spermidine restored these GluA1 post‐translational mechanisms that in the hippocampus are necessary for high memory load processing (Olivito et al., 2016). These findings identify a mechanism by which Spermidine may ameliorate cognition.

### 2.4 TAT‐Beclin 1 rescued GluA1 post‐translational mechanisms in inbred C57BL/6J 16‐month‐old mice

**Figure 5:**
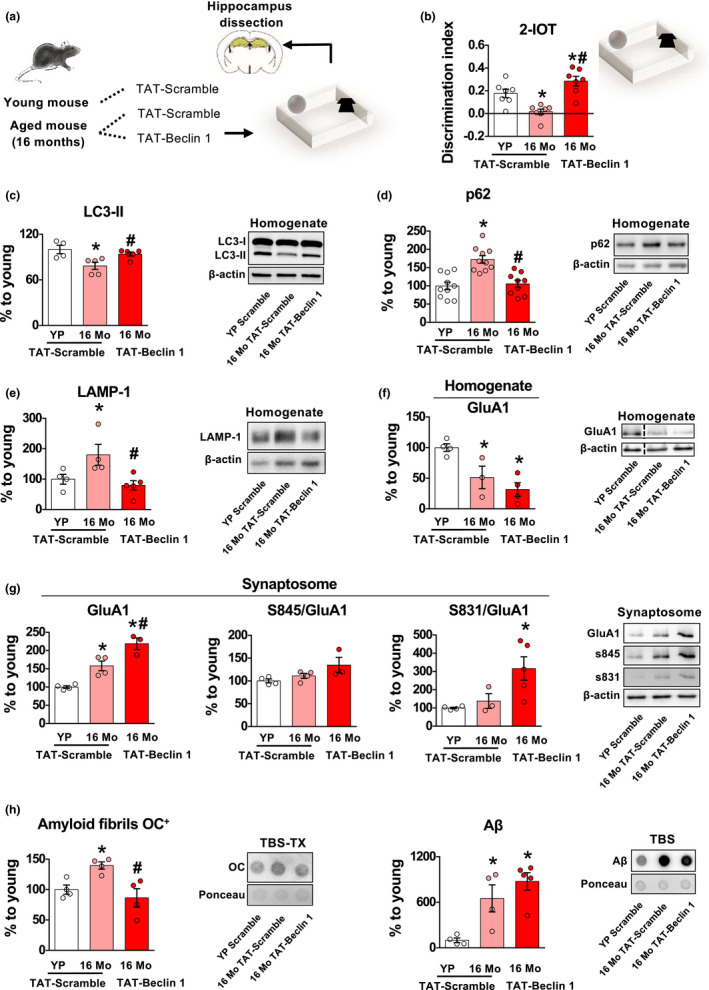
Subchronic treatment with the direct autophagy agonist, TAT‐Beclin 1, rescues GluA1 post‐translational modifications via autophagy‐mediated clearance of amyloid load in 16‐month‐old C57/BL6 memory‐impaired mice. (a) Schematic representation of the experimental procedure. Young (3‐month‐old) were treated with TAT‐Scramble while 16‐month‐old mice were divided in two subgroups treated with TAT‐Scramble or TAT‐Beclin 1. After treatment, they were submitted to the 2‐identical object recognition task (2‐IOT). Hippocampi were dissected immediately at the end of the procedure, and protein extraction was performed on homogenates and synaptosomes fractions. (b) 16‐month‐old (16 Mo) mice showed decreased discrimination index compared to young partners (YP). TAT‐Beclin 1 treatment rescued the memory impairment in 16 Mo mice [*F*_2, 18_ = 14.831, *p* = 0.0002] [YP *n* = 7; 16 Mo *n* = 7; 16 Mo TAT‐Beclin 1 *n* = 7]. (c–e) 16‐month‐old mice showed decreased LC3‐II, accumulation of p62 and LAMP‐1 in the hippocampus, compared to young partners. Subchronic injection of TAT‐Beclin 1 decreased these proteins, suggesting a reactivation of autophagy [LC3‐II: *F*_2, 11_ = 6.51, *p* = 0.013; p62: *F*_2, 26_ = 15.60, *p* = 0.000035; LAMP‐1: *F*_2, 10_ = 5.45, *p* = 0.02]. Representative bands for each condition are presented [YP TAT‐Scramble *n* ≥ 4; 16 Mo TAT‐Scramble *n* ≥ 4; 16 Mo TAT‐Beclin 1 *n* ≥ 5]. (f) GluA1 was reduced in the homogenate fraction of 16‐month‐old mice, compared to YP [*F*_2, 8_ = 9.77, *p* = 0.007]. Representative bands for each condition are presented [YP *n* = 4; 16 Mo *n* = 3; 16 Mo TAT‐Beclin 1 *n* = 4]. (g) GluA1 in the synaptosomal fraction was increased in both TAT‐Scramble and TAT‐Beclin 1 16‐month‐old mice [*F*_2, 8_ = 26.19, *p* = 0.0003], but a tendency to increase the phosphorylation of S845 and a significant increase in the phosphorylation of S831 was induced by TAT‐Beclin 1 treatment [*F*_2, 9_ = 5.87, *p* = 0.02]. Representative bands for each condition are reported [YP *n* = 4; 16 Mo *n* = 4; 16 Mo TAT‐Beclin 1 *n* = 3]. (g) 16‐month‐old mice had increased Aβ in TBS and OC^+^ amyloid fibrils in TBS‐TX fractions, compared to YP. TAT‐Beclin 1 treatment reduced OC^+^ amyloid fibrils but not Aβ [OC^+^ amyloid fibrils: *F*_2, 9_ = 7.14, *p* = 0.01; Aβ: *F*_2, 10_ = 10.52, *p* = 0.003]. Representative dot blots for each condition are presented [YP TAT‐Scramble *n* = 4; 16 Mo TAT‐Scramble *n* = 4; 16 Mo TAT‐Beclin 1 *n* = 4]. Bar charts represent mean ± *SEM*. *p < 0.05 vs. YP TAT‐Scramble, #*p* < 0.05 vs. 16 Mo TAT‐Scramble.

The memory improving effects of Spermidine are in line with recent findings showing that intra‐hippocampal injections of TAT‐Beclin 1, which enhances autophagic/lysosomal degradation through the activation of VPS34‐UVRAG complex (Bartolomeo et al., 2017), rescue fear conditioning memory deficits in 16‐month‐old C57BL/6J mice (Glatigny et al., 2019). This is an inbred mouse line, with little age‐related inter‐individual difference. We decided to test whether we could detect increased amyloid load and impairment in GluA1 functional activation also in this already published advanced model and whether, using a more specific autophagy enhancer, we could rescue them (Figure 5a).

Sixteen‐month‐old mice were tested in the lowest memory load version of the object recognition task (2‐IOT, which is comparable to the 6‐IOT performance in outbred CD1 mice), revealing a strong impairment compared to young mice (3‐month‐old) (Figure 5b), thus suggesting that at 16 months these mice presented an advanced stage of cognitive impairment. Compared to young matched mice, 16‐month‐old C57BL/6J mice showed decreased LC3‐II (as reported in Glatigny et al., 2019) (Figure 5c), accumulation of p62 (Figure 5d) and LAMP‐1 in the hippocampus (Figure 5c–e), therefore suggesting reduced autophagosome formation and degradative capacity. Systemic TAT‐Beclin 1 injections, at a dose which stimulates autophagy (Figure 5c–e), effectively rescued memory impairment, in line with the previous report (Glatigny et al., 2019) (Figure 5b). Interestingly, 16‐month‐old C57BL/6J mice also had reduced expression of GluA1 in the homogenate fraction (Figure 5f) and accumulation in the synaptosomes (Figure 5g). Also in this case, no increase in the phosphorylation of S831 and S845 (Figure 5g) was observed, similar to the CD1 *Impaired* model. At the same time, 16‐month‐old C57BL/6J mice showed increased intra‐ and extracellular OC^+^ amyloid fibrils and intracellular Aβ (Figure 5h and Figure S6a,b), resembling the 12‐month *Impaired* phenotype. Interestingly, chronic treatment with TAT‐Beclin 1, although did not rescued the decrease of GluA1 expression in the homogenate, increased synaptosomal GluA1 expression and phosphorylation of both S845 and S831 (Figure 5e,f). Furthermore, it reduced TBS‐TX OC^+^ amyloid fibrils (Figure 5h) but had no effect on both Aβ in TBS and TBS‐TX and on OC^+^ amyloid fibrils in the TBS (Figure 5h; Figure S6a,b). These findings are in line with previous work showing the role of autophagy/lysosomal degradation of amyloid load and further supports the idea that pharmacological improvement in the autophagic/lysosomal degradative capacity allows the activation of memory‐induced post‐translational modification of GluA1 necessary for hippocampal‐dependent memory.

## 3 DISCUSSION

In this study, we show that Spermidine, as well as the more specific autophagy enhancer TAT‐Beclin 1, by favouring autophagic/lysosomal degradation of amyloid fibrils, re‐establishes the functional activation of GluA1 receptors required for hippocampal‐dependent memory. This finding provides the first evidence of a mechanism by which Spermidine restores synaptic functions in a model of MCI in middle‐age subjects.

There is much attention in the literature for the identification of early markers of dementia, which is crucial for early diagnosis. In this study, we present a novel model of MCI in rodents in which, using the 6‐DOT/6‐IOT, it is possible to: a. identify subtle cognitive deficits in condition of high load, while performance is still preserved in conditions of low load; b. discriminate between impaired and cognitively preserved subjects; c. predict memory performance tested 6 months later.

We have previously reported that knock‐in mutant mice lacking both S845 and S831 phosphorylation sites on the GluA1 subunit are impaired in the 6‐DOT, but not in the 6‐IOT (Olivito et al., 2016). In addition, here we report that the 6‐DOT induces a significant synaptic enrichment of GluA1 accompanied by increased phosphorylation (Figure S5A,B), possibly to sustain the high memory load of the task. In *Impaired* mice, this enrichment occurs as a nonfunctional compensatory mechanism. This is line with a previous study showing that impaired performance in the hippocampal‐dependent spatial water maze task pinpointed impaired GluA1 phosphorylation in 24‐month‐old rats (Yang et al., 2015). Hippocampal GluA1 phosphorylation increases the current amplitude, regulates the trafficking and composition of AMPA receptors (Oh, Derkach, Guire, & Soderling, 2006) and is necessary for performing the 6‐DOT as well as other types of hippocampal‐dependent memory tasks (Olivito et al., 2016). Thus, impaired AMPA receptors post‐translational mechanisms seem to underlie vulnerability to age‐ and hippocampal‐dependent memory deficits.

Although mice have been suggested to be less prone to produce Aβ plaques (Kimura, Hata, & Suzuki, 2016), few studies specifically addressed this issue in ageing wild‐type animals. A recent report identified widespread Aβ‐positive extracellular aggregates in the brain of 15‐month‐old C57/BL6 wild‐type mice (Ahlemeyer, Halupczok, Rodenberg‐Frank, Valerius, & Baumgart‐Vogt, 2018), and similar findings have been obtained with p‐α‐syn (Takahashi, Ohsawa, Shirasawa, & Takahashi, 2016). In line with these findings, *Impaired* mice showed a blockade of autophagy and accumulation of Aβ and α‐syn fibrils (OC^+^) similarly to what has been previously observed in a genetic animal model of AD (Rocchi et al., 2017), synucleinopathy (Giordano et al., 2018) and normal ageing mice (Ahlemeyer et al., 2018; for review see Cuervo et al., 2005); α‐syn fibrils were found to accumulate in enlarged lysosomes suggesting a negative feedback loop between the accumulation of amyloid fibrils and impaired autophagic/lysosomal degradative capacity (Nixon et al., 2005; Yamamoto et al., 2019). To our knowledge, this is the first mouse model of MCI for middle‐age animals that recapitulates human studies showing that MC decline is an early marker of cognitive function prodromal to AD development (Belleville et al., 2007).

Despite an enormous amount of evidence on the beneficial effects of Spermidine for healthy ageing, the synaptic mechanisms by which it improves cognition have been not completely addressed. Spermidine has been shown to regulate learning and memory in many animal models (Fruhauf et al., 2015; Guerra et al., 2011; Signor, Mello, Porto, Ribeiro, & Rubin, 2014), and this action has been associated with the regulatory role on polyamines on NMDA and calcium permeable receptors (Williams et al., 1991) and to the inhibition of the RAMP‐UP of synapses occurring in ageing, a phenomenon for which active zones of the synapse scale up their size with ageing (Bhukel et al., 2017; Gupta et al., 2016). We expand on these findings showing that Spermidine favours the lysosomal clearance of amyloid fibrils including α‐syn and Aβ and rescues the memory load‐dependent hippocampal post‐translational modifications of GluA1 in *Impaired* mice. We report here that this effect is likely mediated by the activation of TFEB in the hippocampus, which is a master gene regulating lysosomal biogenesis (Sardiello et al., 2009). This is in line with recent findings showing that Spermidine has direct transcriptional regulation effects on TFEB and its activation (Zhang et al., 2019). This finding has high translational relevance as genetic‐mediated overexpression of TFEB has been shown to rescue behavioural and neuropathological disease progression in animal models of synucleinopathy and AD (Decressac et al., 2013; Polito et al., 2014). Although TAT‐Beclin 1 did not fully rescue the increased extracellular amyloid fibrils and Aβ burden in these older animals, it efficiently reduced the intracellular amyloid fibrils burden and re‐established the task‐dependent functional activation of AMPA receptors in the hippocampus. It is highly unlikely that the ameliorative effects of Spermidine on memory and GluA1 phosphorylation were mediated by its direct activation of PKA and PKC (Guerra et al., 2011), as they are prevented by concurrent administration of the selective autophagy inhibitor, 3‐MA. This agonist/antagonist pharmacological experiment clearly showed that the pro‐cognitive and synaptic effects of Spermidine were mediated by autophagy/lysosomal pathway activation.

Emerging evidence suggests that autophagy directly regulates memory, synaptic plasticity and AMPA (GluA1 and GluA2 subunits) in physiological conditions (Hylin et al., 2018; Shehata, Matsumura, Okubo‐Suzuki, Ohkawa, & Inokuchi, 2012). In ageing, data converge on suggesting that hippocampal GluA1 post‐transcriptional modifications necessary for learning are impaired by the increased burden of amyloid load and impaired autophagy. Previous findings clearly showed that soluble oligomeric Aβ induces dephosphorylation of GluA1 at S845 (Guntupalli et al., 2016; Minano‐Molina et al., 2011), which is not only involved in GluA1 insertion at the synapse, but also in AMPA receptors recycling (Ehlers, 2000; Man, Sekine‐Aizawa, & Huganir, 2007), which parallels the dephosphorylated GluA1 accumulated at the synaptic site in memory‐*Impaired* mice. We provide here conclusive evidence that boosting the autophagic/lysosomal degradative capacity in cognitively impaired ageing mice, with systemic administrations of agonist drugs, improves the degradation of amyloid fibrils and rescues the post‐translational modification of glutamate receptors.

There is an enormous attention towards autophagy agonists to counteract the age‐related onset of dementia, and these findings have high translational relevance considering a recent clinical trial showing that Spermidine ameliorates memory performance in a small cohort of subjects with MCI compared to placebo‐treated subjects (Wirth et al., 2018).

To our knowledge, our study provides the first identification of a mechanism by Spermidine treatment, as well as other autophagy agonists, and improves memory in middle‐age cognitively impaired subjects. By linking amyloid load to AMPA receptors functions, our data posit neurodegenerative diseases along a *continuum* from healthy to pathological ageing, leading to dementia. A Spermidine‐rich diet may therefore constitute a quite easy and safe strategy to counteract ageing and age‐related memory decline, which is one of the most challenging need to limit the “epidemic” incidence of ageing‐related and genetic forms of dementia.

## 4 MATERIALS AND METHODS

### 4.1 Subjects

CD1 outbred male mice were generated from internal colonies by founders obtained by Charles River (Como, Italy). Mice were group‐housed with food and water ad libitum on a 12 hr light/dark cycle. Behavioural tests were performed during the light phase. The age of mice ranged from 3 to 12 to 18‐month‐old.

Three and 16 months C57BL/6J male mice were obtained from Janvier Laboratory stock. All procedures related to animal care and treatments were conducted in accordance with the European Communities Council directives and Italian laws on animal care. All experimental protocols were approved by Italian Ministry of Health.

### 4.2 Experimental design and behavioural procedures

All experiments were designed to evaluate biochemical changes (glutamate receptors expression and phosphorylation, amyloid load and autophagy) under test challenge.

High memory load was tested in the 6 different objects task (6‐DOT) and as control task in low memory load conditions the 6 identical objects task (6‐IOT) was used, as it has the same number of elements to explore but all identical copies (low information load), using behavioural procedures identical to what we previously described (Olivito et al., 2016; Sannino et al., 2012). Mice exploring objects for less than 5 seconds in the study phase were excluded from the statistical analysis. Mouse behaviour was analysed using a video‐tracking system (Anymaze, Stoelting, USA) by a trained experimenter. For the 6‐DOT/6‐IOT data analysis, total object exploration (for the study phase) and New % exploration (for the test phase) were considered.

We conducted three main experiments:

1. a *longitudinal study*;
2. a *study on Spermidine action and mechanisms on ageing*;
3. a *study on TAT*‐*Beclin 1 subchronic treatment on ageing*.

In all the experiments, mice were habituated to the testing conditions.

*Experiment 1*: all subjects were tested at 12‐month‐old in the 6‐DOT and in the 6‐IOT one week apart. The percentage of the exploration of the new object (New % Exploration) was calculated as: [(New object exploration/Σ objects exploration) * 100].

Memory performance of mice was individually analysed for assignment in two subgroups: *Preserved* or *Impaired*. The following criterion was used:

*Preserved*: New > [(Mean Familiar) + (SD * 1.5)];
*Impaired*: New < [(Mean Familiar) + (SD * 1.5)].

Mice from the *Preserved* and *Impaired* subgroups were additionally tested at 18 months in the 6‐DOT or the 6‐IOT, respectively.

*Preserved* mice at 12 months were considered *Stable* at 18 months if after the 6‐DOT the criterion for *Preserved* was confirmed or *Declined* if the criterion for *Impaired* was fit.

*Impaired* mice at 12 months mice were considered *Stable* if after the 6‐IOT the criterion for *Preserved* was confirmed or *Declined* if the criterion for *Impaired* was fit.

*Experiment 2*: a group of 12‐month‐old mice was tested in the 6‐DOT and subdivided into *Preserved* and *Impaired* as before. Mice from the *Impaired* group were selected and submitted to a subchronic treatment with: vehicle (PBS), Spermidine (50 mg/kg), 3‐MA (10 µM) or Spermidine+ 3‐MA. *Preserved* mice were treated with vehicle. The subchronic treatment consisted in 1 month of 3 intraperitoneal (ip) injections *per* week. After this period, all mice were tested again in the 6‐DOT with a completely different set of objects. Subsequently, they were sacrificed and the brain was extracted for hippocampal dissection or put in paraformaldehyde 4% in phosphate‐buffered saline (PBS) followed by 30% sucrose solution for immunofluorescence procedures. Data for this experiment were replicated on mice from two different animal facilities.

*Experiment 3*: 3‐ or 16‐month‐old C57BL/6J mice were injected with 450 µg of TAT‐Beclin 1 (cat # 5.06416.0001, Merck; dissolved in PBS) or TAT‐Scramble (cat # 5.31038.001, Merck; dissolved in PBS) by ip injection each day for 17 days (Glatigny et al., 2019). Mice were then subjected to object recognition task with two identical objects (2‐IOT), which is comparable to the 6‐IOT in outbred CD1 mice. Briefly, during the training phase, mice were allowed to explore two identical objects. During the testing phase, objects were replaced with a familiar object and a novel one. The discrimination index was calculated as follow: (time spent exploring the new object ‐ time spent exploring the familiar object)/(total time spent exploring both objects). After the test, mice were sacrificed. Mouse brains were dissected, snap frozen and processed for biochemical analysis.

### 4.3 Synaptosome preparation, Western blotting, TBS/TBS‐TX protein extraction and native Dot Blot

We used standard procedures already described in previous studies (Pignataro et al., 2019). See Appendix S1 for details.

### 4.4 Immunofluorescence

Immunofluorescence was performed as previously described (Giordano et al., 2018). See Appendix S1 for details.

### 4.5 Statistical analysis

The percentage of *Stable* and *Declining* animals of the *longitudinal study* was determined and compared with chi‐squared test. Before applying the ANOVA tests, data distribution normality was tested with the Kolmogorov–Smirnov test. One‐way ANOVA was used to evaluate the behavioural, biochemical and immuhistochemical differences between *Preserved* and *Impaired* mice, followed with Dunnett's post hoc test vs. *Preserved* group when appropriate. A two‐way ANOVA [pretreatment (2 levels: saline and SPD) and treatment (2 levels: saline and 3‐MA)] was used to analyse the behavioural and biochemical effects of pharmacological treatments on *Impaired* mice, followed by a Tukey's post hoc analysis if appropriate. Statistical significance was set at *p* < 0.05.

## CONFLICT OF INTEREST

The authors have no financial disclosures, nor conflict of interest to declare.

## AUTHOR CONTRIBUTIONS

M.D.R. and G.T. planned and performed experiments, analysed the data and generated a first draft of the manuscript; M.T. helped to perform the experiments; S.M. and M.R. performed TAT‐Beclin 1 treatment; A.P. performed part of the immunofluorescence analyses; S.M. helped with the immunofluorescence analyses; N.C. helped to acquire immunofluorescence images; F.G. and S.M. contributed to write the manuscript; C.S. contribute to interpret the autophagy‐related experiments and to write the final version of the manuscript, M.A.T., A.M. and F.O. contributed to write the manuscript and co‐supervised experiments; E.D.L. conceived the study planned, supervised the experiments, performed statistical analysis and wrote the manuscript.

## Supporting information

Supplementary Material

## Data Availability

The data that support the findings of this study are available from the corresponding author upon reasonable request.
