# Supplementary material for: Mechanisms by which autophagy regulates memory capacity in ageing
Source: Aging Cell. 2020 Jul 30;19(9):e13189. doi: 10.1111/acel.13189 (PMC7511873; doi:10.1111/acel.13189)
Supplement: Supplementary file 1 — Supplementary Material [file ACEL-19-e13189-s001.docx]

**Supporting Information**

**Experiments**

The following supplementary experiments were performed:

1. A supplementary young-aged comparison study was performed. 12-month-old mice were coupled to a young partner (YP), 3-month-old, that was allowed to explore exactly the same time as the aged partner (AP) during the study phase. Aged mice were divided in *Preserved* or *Impaired* following this criterion:

*Preserved*: New > [(Mean Familiar) + (SD*1.5)];

*Impaired*: New < [(Mean Familiar) + (SD*1.5)].

New % Exploration was analysed using a one-way ANOVA (YP *vs. Preserved* and YP *vs. Impaired*).

At the end of the task, mice were sacrificed, brains were extracted for biochemical analysis (after hippocampus dissection) or were put in paraformaldehyde 4% in phosphate buffer saline (PBS) followed by 30% sucrose solution for immunofluorescence procedures. Data for this experiment were replicated on mice from three different animal facilities.

For biochemical analysis, hippocampal homogenate or synaptosomes were processed for western blot.

1. 3-month-old mice were injected with a single dose of Spermidine (50 mg/kg) or 3-MA (10 µM). After the injection (2 or 4 hours respectively), mice were sacrificed and brains were extracted for biochemical analysis. Hippocampal homogenate or synaptosomes were processed for western blot. Optical density was quantified with ImageJ software (NIH) and analysed with unpaired t-test.
2. 3-month-old mice were submitted to 6-DOT, while control group were submitted only to habituation to the experimenter. At the end of the procedure, mice were sacrificed and brains were extracted for biochemical analysis. Hippocampal homogenate or synaptosomes were processed for western blot. Optical density was quantified with ImageJ software (NIH) and analysed with unpaired t-test.

**Materials and methods**

**Synaptosome preparation and western blotting**

Primary antibodies used were: anti-GluA1 (1:500, ab31232, Abcam), anti-GluA2 (1:500, ab20673, Abcam), anti-p-S845 (1:500, 36-8300, Invitrogen), anti-p-S831 (1:500, AB5847, Millipore), anti-LC3 (1:3000; NB100-2220, Novus Bio), anti-p62 (1:500; H00008878-M01; Tebu-Bio), anti-LAMP-1 (1:500; sc-19992; Santa Cruz), anti-TFEB (1:1000; A303-673A; Bethyl); anti-GluA3 (1:1000; MAB5416; Millipore), anti-PSD95 (1:1000; 3450, Cell Signalling), anti-Synaptophysin (1:2000; 5461; Cell Signalling), anti-catalytic PKA α subunit (1:1000, sc-28316, Santa Cruz), anti-regulatory PKA RIIα subunit (1:1000, HPA045703, Sigma) and anti-β-actin (1:5000, MAB1501, Millipore) as loading control. The appropriate secondary antibody was incubated (1:5000, BioRad) for 1 hr at room temperature. Immunoreactivity was detected by chemiluminescence and bands were quantified by densitometry using ImageJ software (NIH).

**TBS/TBS-TX protein extraction and native Dot blot**

Sequential extraction on TBS and TBS-TX was performed as previously described ([Pignataro et al., 2019](#_ENREF_1)). Samples were homogenized in Tris-buffered saline (TBS: 20 mM Tris HCl, 150 mM NaCl, pH 7.4, v/w 2:1) and ultra-centrifuged in a TLA 100.4 Rotor (Beckman Coulter) at 65000 rpm for 60 minutes. The supernatant represents the TBS extract. Then, the pellet was homogenized in TBS containing 1% Trition-X-100 (TBS-TX, v/w 2:1) and centrifuged using the same conditions used for TBS extracts. The supernatant represents the TBS-TX extract. Proteins from TBS and TBS-TX fractions were then used for Dot blot. 150 ng of homogenates from TBS and TBS-TX fractions were spotted on a nitrocellulose membrane 0.22 μm and then incubated with following primary antibodies: anti-Aβ Clone 295F2 (1:1000, 218 721, Synaptic System) and anti-OC (1:1000, AB2286, Millipore). Ponceau S staining was used as loading control. Immunoreactivity was detected by chemiluminescence and dots were quantified by densitometry using ImageJ software (NIH).

**Immunofluorescence procedure**

30 µm hippocampal slices were cut with a cryostat (Leica). Slices were incubated with primary antibodies against: LAMP-1 (1:300, sc-19992, Santa Cruz), p-α-syn (1:100, ab51253, Abcam), p62 (1:500, H00008878-M01, Tebu-Bio) or NeuN (1:300, ABN90, Merck Millipore) after a blocking step with PBS-Triton X-100 0.3% and 5% normal goat serum (NGS). Two batches of slices were incubated with primary antibody against PSD95 (1:100, ADI-VAM-PS002-E, Enzo Life Sciences, Inc.) or both PSD95 and GluA1 (1:400; ab31232, Abcam) without the blocking step. Slices were subsequently washed with PBS and incubated for 2 h with the proper secondary antibodies (1:300, donkey anti-rabbit Alexa-Fluor® 568, ab175470; 1:300, goat anti-guinea pig Alexa-Fluor® 568, ab175714; 1:300, goat anti-rat Fluorescein conjugated, AP136F). For nuclear counterstaining slices were incubated with DAPI (1:1000, 10 min, D1306, Invitrogen) before mounting and inclusion with a Mowiol 4-88 (Sigma Aldrich) solution.

Images of NeuN were acquired with a confocal microscope (Zeiss, LSM700 Axio Observer) at 20x magnification. Images of LAMP-1/p-62 and LAMP-1/p-α-syn immunostaining were acquired with a confocal microscope (Olympus, IX83) at 40x magnification. Images for PSD95 alone were acquired with a confocal microscope (Olympus, IX83) at 60x and 2x digital zoom. Images of PSD95/GluA1 colocalization were acquired as z-stacks (10 sections of 0.5 μm) using the super resolution module Airyscan2 at a confocal microscope (Zeiss, LSM900 Axio Observer) at 60x with 2x digital zoom. All images were acquired with a 1024 x 1024 pixel resolution. For all the experiments 2-5 slice *per* mouse and 3-4 mice *per* group were acquired and analysed.

The number of NeuN^+^ neurons per slice were determined in the CA1, CA3 and DG of the hippocampus with the use of FIJI (ImageJ, NIH, USA); data were then summed per CA1, CA3 and DG of each slice, averaged per slices of each experimental condition and expressed as percentage of young.

The number of LAMP-1^+^, p62^+^ and p-α-syn^+^ spots were identified and measured in the CA3 of the hippocampus using Imaris 7.1.1 software (Oxford Instruments). Each image was sampled with 4 regions of interest (ROIs: 70x70 µm) avoiding DAPI-labelled cell bodies, and spots were automatically counted with settings of diameter as follows: 1 µm and 3 µm for small and large LAMP-1, respectively: 1 µm for p62; 2.5 µm for p-α-syn; 0.2 µm for PSD95 and GluA1. For PSD95/GluA1 colocalization Imaris 7.1.1 software automatically detects and counts PSD95^+^ and GluA1^+^ spots and identifies colocalizations as overlapping signals. PSD95/GluA1 colocalization analysis was performed on 4 ROIs (10x10 µm).

ROIs were analysed establishing a detection threshold constant within each measurement. The number of spots were averaged per slices of each experimental condition and expressed as percentage of young or pMC.

**Quantitative real-time PCR**

GluA1 gene was examined using the following primers: forward 5’-ACTTACCGATTCTGTTCCCA-3’; reverse 5’-ATTGGATGTGTCAACGGGAA-3’. Hypoxanthine phosphoribosyl transferase (HPRT) gene was chosen as a housekeeping gene for normalization using the following primers: forward 5’-GACTGATTATGGACAGGACT-3’; reverse 5’-ATTGTAATCCAGCAGGTCAG-3’. The delta Ct ((ΔCt) threshold cycle) was determined for each gene relative to HPRT.

**Supplementary Reference**

Pignataro, A., Meli, G., Pagano, R., Fontebasso, V., Battistella, R., Conforto, G., . . . Middei, S. (2019). Activity-Induced Amyloid-beta Oligomers Drive Compensatory Synaptic Rearrangements in Brain Circuits Controlling Memory of Presymptomatic Alzheimer's Disease Mice. *Biol Psychiatry, 86*(3), 185-195. doi: 10.1016/j.biopsych.2018.10.018

**Tables**

| Experiment | Memory Capacity  12 MONTHS | Study phase  (6-DOT) | Study phase  (6-IOT) | Memory Capacity  18 MONTHS | Study phase  (6-DOT) | Study phase  (6-IOT) |
| --- | --- | --- | --- | --- | --- | --- |
| Longitudinal Study | *Preserved* | 129.6±10.2 | 30.8±1.5 |  |  |  |
|  | *Impaired* | 107.1±12.5 | 29.0±2.2 |  |  |  |
|  | *Preserved* |  |  | *Stable* | 126.8±12.3 | 30.8±2.1 |
|  |  |  |  | *Declined* | 76±35.5 | 26.7±7.3 |
|  | *Impaired* |  |  | *Stable* |  | 17.0±2.0 |
|  |  |  |  | *Declined* |  | 23.3±2.5 |

**Table S1.** No differences were revealed in the total time of exploration during the study phase at 12 months in the 6-DOT and in the 6-IOT. *Preserved* mice were tested again at 18 months and showed no differences in the total exploration time nor in the 6-DOT neither in the 6-IOT. Similarly, *Impaired* mice were tested directly in the 6-IOT at 18 months, showing no difference in the object exploration time at study phase [F_1,8_=1.438; p<0.2648]. Data represent mean ± S.E.M.

| Experiment | Group | Study phase  (6-DOT) |
| --- | --- | --- |
| Young-Aged Comparison Study | YP (pMC) | 148.1±12.9 |
|  | pMC | 152.3±10.5 |
|  | YP (iMC) | 126.5±14.8 |
|  | iMC | 126.2±15.0 |

**Table S2.** Object exploration during the study phase of pMC, iMC and their respective YP, showed no significant difference. Data represent mean ± S.E.M.

| Experiment | Group | Study phase  (6-DOT- TEST) | Study phase  (6-DOT- RETEST) |
| --- | --- | --- | --- |
| Spermidine Subchronic Treatment | pMC Vehicle | 155.625±9.329 | 133.613±17.913 |
|  | iMC Vehicle | 151.583±27.680 | 108.050±21.697 |
|  | iMC  + Spermidine | 174.550±9.528 | 87.875±7.688 |
|  | iMC + 3-MA | 163.060±13.202 | 69.210±7.304 |
|  | iMC  + Spermidine  + 3MA | 125.160±13.932 | 86.220±16.190 |

**Table S3.** Objects exploration during the study phase of the test and retest after subchronic treatment of Spermidine, 3-MA or Spermidine + 3-MA. Data represent mean ± S.E.M.

| Experiment | Group | Habituation phase  (6-DOT- TEST) | Habituation phase  (6-DOT- RETEST) |
| --- | --- | --- | --- |
| Spermidine Subchronic Treatment | pMC Vehicle | 38.020±2.893 | 33.038±2.322 |
|  | iMC Vehicle | 34.644±4.735 | 30.084±4.735 |
|  | iMC  + Spermidine | 40.445±5.088 | 26.477±3.131 |
|  | iMC + 3-MA | 33.221±3.719 | 18.367±1.773 |
|  | iMC  + Spermidine  + 3MA | 37.269±5.644 | 27.496±4.163 |

**Table S4.** Total distance travelled during the habituation phase of the test and retest after subchronic treatment of Spermidine, 3-MA or Spermidine + 3-MA. No significant differences were present between groups. Data represent mean ± S.E.M.

| Experiment | Group | Study phase  (2-IOT) |
| --- | --- | --- |
| TAT-Beclin 1 Subchronic Treatment | 3 months TAT-Scramble | 36±2.469 |
|  | 16 months TAT-Scramble | 36.143±5.958 |
|  | 16 months TAT-Beclin 1 | 33±6.576 |

**Table S5**. Mean Total objects exploration during the study phase of the 2-IOT after subchronic treatment of TAT-Scramble or TAT-Beclin 1 in 3 and 16 month-old mice. Data represent mean ± S.E.M.

**Figures**

**
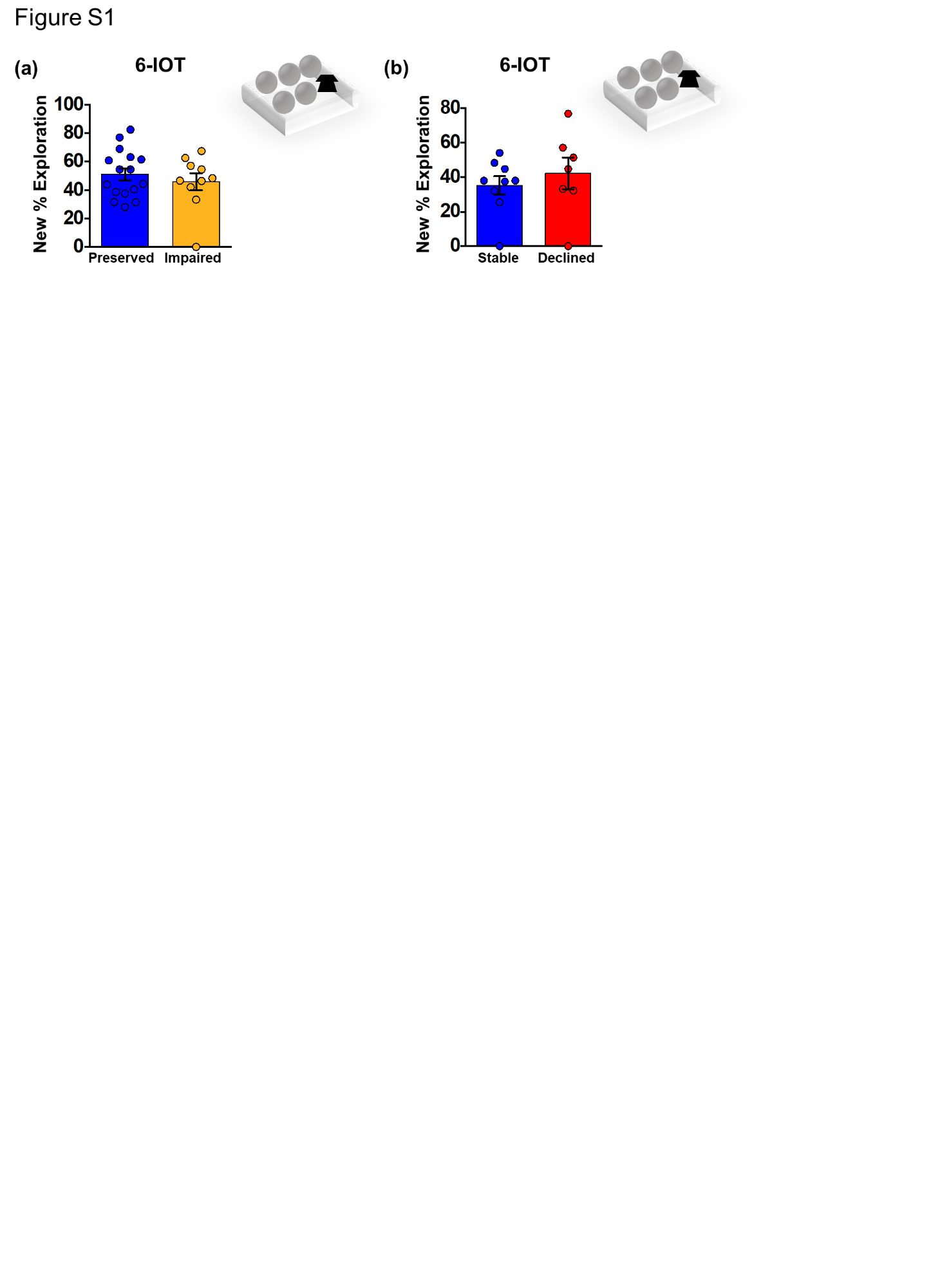
**

**(a).** The percentage exploration of the new object in the 6-IOT (low memory load task) for *Preserved* and *Impaired* mice at 12-month-old. Data show no difference between groups in the task with low memory load condition. Bar charts represent mean ± S.E.M.

**(b).** Mice belonging to the *Preserved* group (showing no impairment in the 6-DOT at 12 months) did not decline their performance in the 6-IOT at 18 months. Bar charts represent mean ± S.E.M.

**
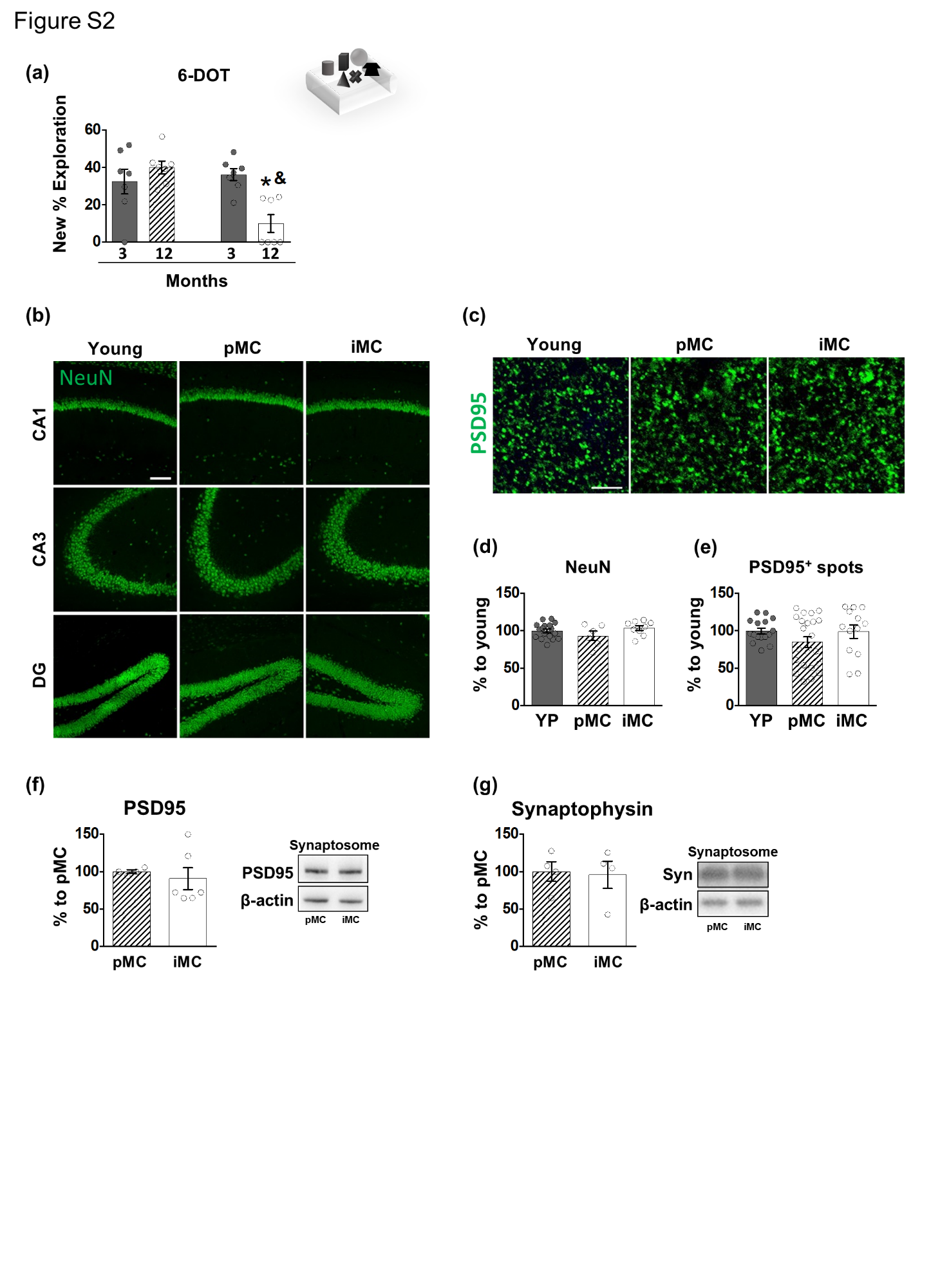
**

**(a).** Percentage exploration of the new object in all 12-month-old *Preserved* (pMC) and *Impaired* (iMC) compared to their respective young-partner (YP) [Age F_1,24_=3.834, p=0.0619; Test F_1,24_=7.656, p=0.0107; Age x Test F_1,24_=12.511, p=0.0017]. Bar charts represent mean ± S.E.M. *p<0.05 *vs* 12 months pMC; & p<0.05 *vs* 3 months.

**(b-d).** Representative images for NeuN immunofluorescence for each subregion of the hippocampus analysed in young, pMC and iMC groups. Corresponding quantification (**d**) showed no differences in the number of neurons in none of the groups [YP n=17; pMC n=6; iMC n=9]; scale bar 100 µm.

**(c-e).** Representative images for PSD95 immunofluorescence in the hippocampus of young, pMC and iMC groups. Corresponding quantification (**e**) showing no differences in the number of PSD95^+^ spots in none of the groups [YP n=16; pMC n=22; iMC n=13]; scale bar 5 µm. Bar charts represent mean ± S.E.M.

**(f).** Levels of PSD95 in pMC and iMC. Representative bands for each condition are presented [pMC=4; iMC=6]. Bar charts represent mean ± S.E.M.

**(g).** Levels of Synaptophysin in pMC and iMC. Representative bands for each condition are presented [pMC=4; iMC=4]. Bar charts represent mean ± S.E.M.

**
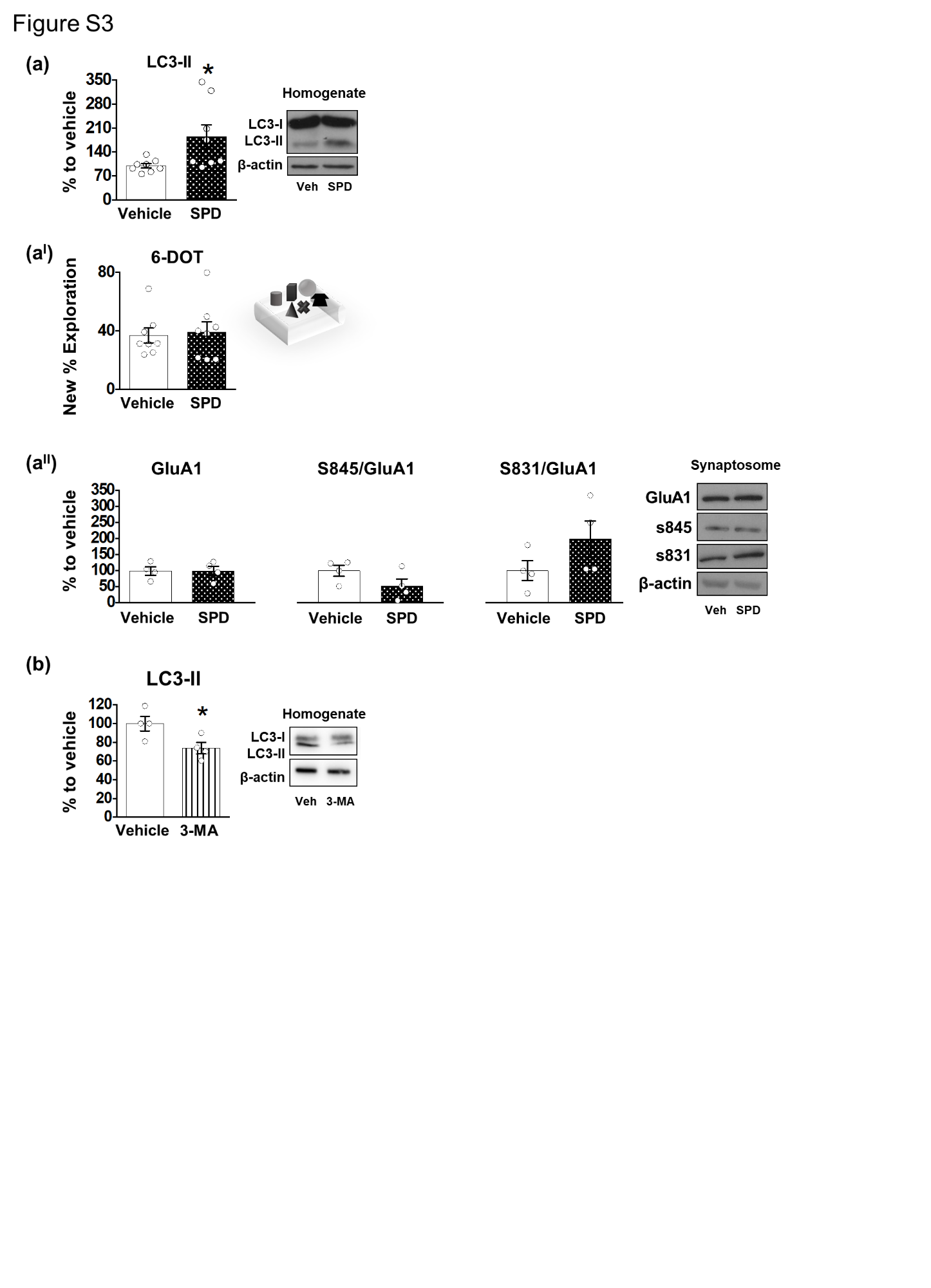
**

**(a).** LC3-II levels after acute injection of Spermidine (SPD) [p=0.0316]. Representative bands for each condition are presented [Vehicle n=8; SPD n=8]. Bar charts represent mean ± S.E.M. *p<0.05 *vs.* vehicle, between groups.

**(a^I^).** Percentage exploration of the new object after acute injection of Spermidine [Vehicle n=8; SPD n=8]. Bar charts represent mean ± S.E.M.

**(a^II^).** GluA1 levels and phosphorylation of S845 and S831 after acute injection of Spermidine. Representative bands for each condition are presented [Vehicle n=4; SPD n=4]. Bar charts represent mean ± S.E.M.

**(b).** LC3-II levels after acute injection of 3-MA [p=0.0381]. Representative bands for each condition are presented [Vehicle n=4; 3-MA n=4]. Bar charts represent mean ± S.E.M. *p<0.05 *vs.* vehicle, between groups.

**
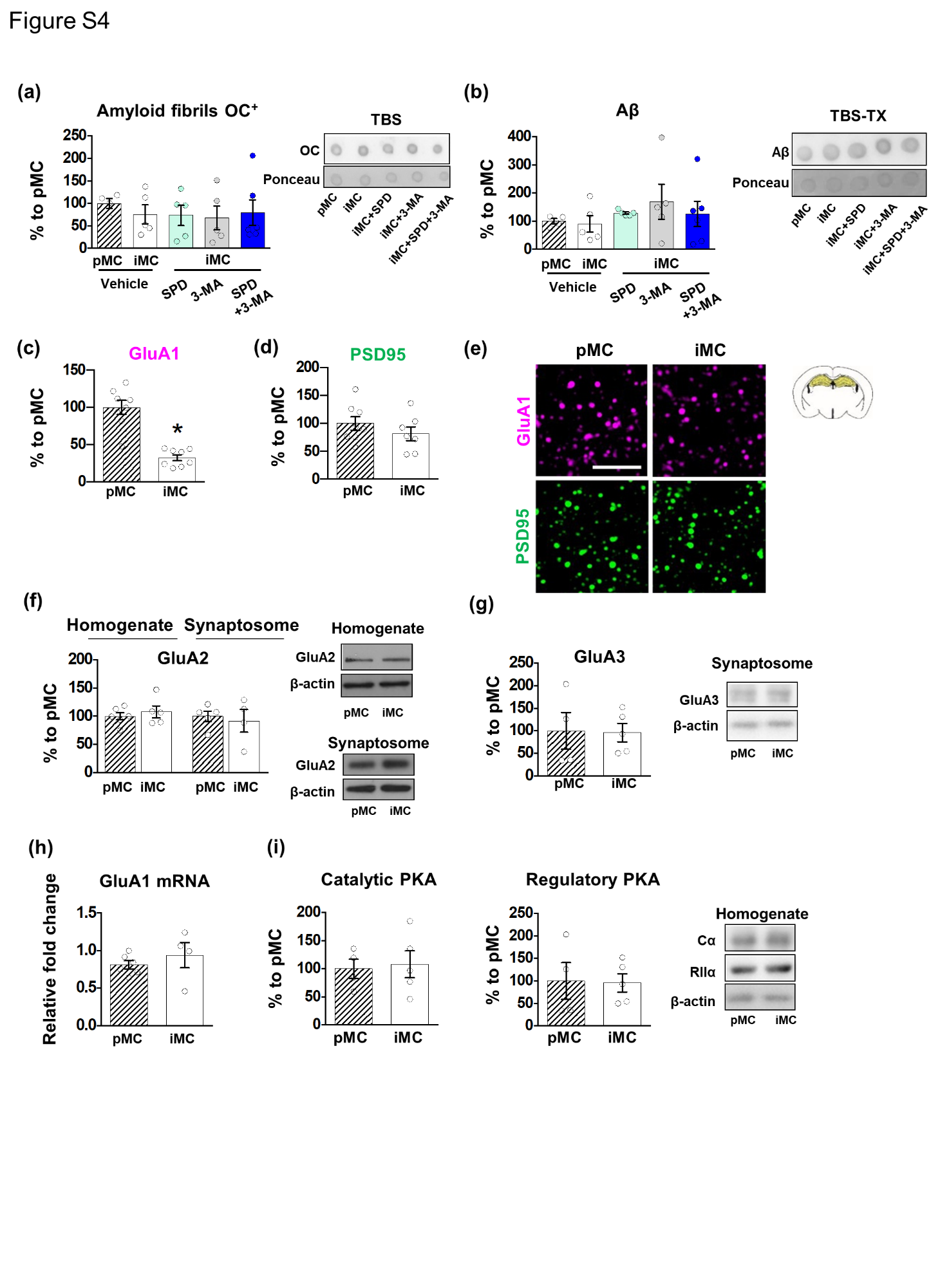
**

**(a-b).** OC^+^ amyloid fibrils levels in TBS extracts and Aβ levels in TBS-TX extracts in pMC and iMC treated with vehicle and iMC subchronically treated with Spermidine (SPD), 3MA and Sperminidine+3MA. Representative dot blots for each condition are presented [pMC n=4; iMC vehicle n=5; iMC SPD n=5; iMC 3-MA n=5; iMC SPD+ 3-MA n=6]. Bar charts represent mean ± S.E.M.

**(c-d).** GluA1^+^ spots (**c**) were significantly lower in iMC mice [p<0.0001], while no differences were found for PSD95^+^ spots (**d**) [pMC n=8; iMC n=7]. Bar charts represent mean ± S.E.M.

**(e).** Representative images of GluA1 and PSD95 immunofluorescence. Scale bar: 5 µm.

**(f).** GluA2 protein levels between pMC and iMC subjects in the homogenate and in the synaptosomes [homogenate: pMC n=6; iMC n=5; synaptosome: pMC n=5; iMC n=4]. Bar charts represent mean ± S.E.M.

**(g).** Levels of GluA3 in pMC and iMC. Representative bands for each condition are presented [pMC n=4; iMC n=5]. Bar charts represent mean ± S.E.M.

**(h).** Relative fold change of GluA1 mRNA in pMC and iMC population, showing no change at the level of GluA1 transcripts [pMC n=6; iMC n=4]. Bar charts represent mean ± S.E.M.

**(i).** Levels of catalytic (Cα) and regulatory (RIIα) subunits PKA levels in pMC and iMC. Representative bands for each condition are presented [pMC n=4; iMC n=5]. Bar charts represent mean ± S.E.M.

**
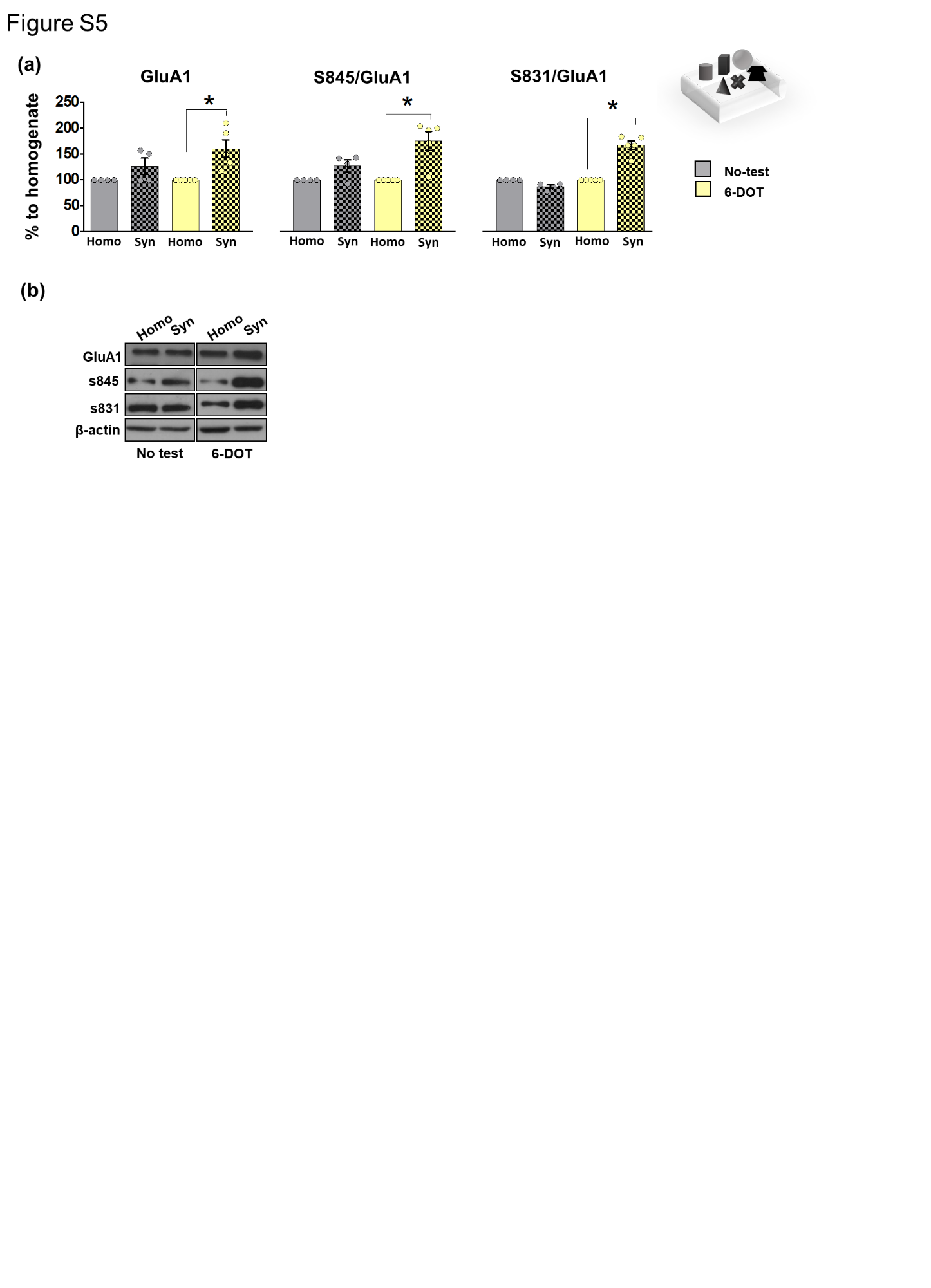
**

**(a-b).** The exposition to the 6-DOT (high memory load) cause a synaptic enrichment of GluA1 [homo vs syn: p=0.0082] and increases phosphorylation of S845 [p=0.0032] and S831 [homo vs syn: p<0.0001] in the same fraction in young mice [No-test: Homogenate n=4; Synaptosome n=4; 6-DOT: Homogenate n=5; Synaptosome n=5]. Panel b shows representative bands. Bar charts represent mean ± S.E.M. *p<0.05 *vs.* homogenate, between groups.

**
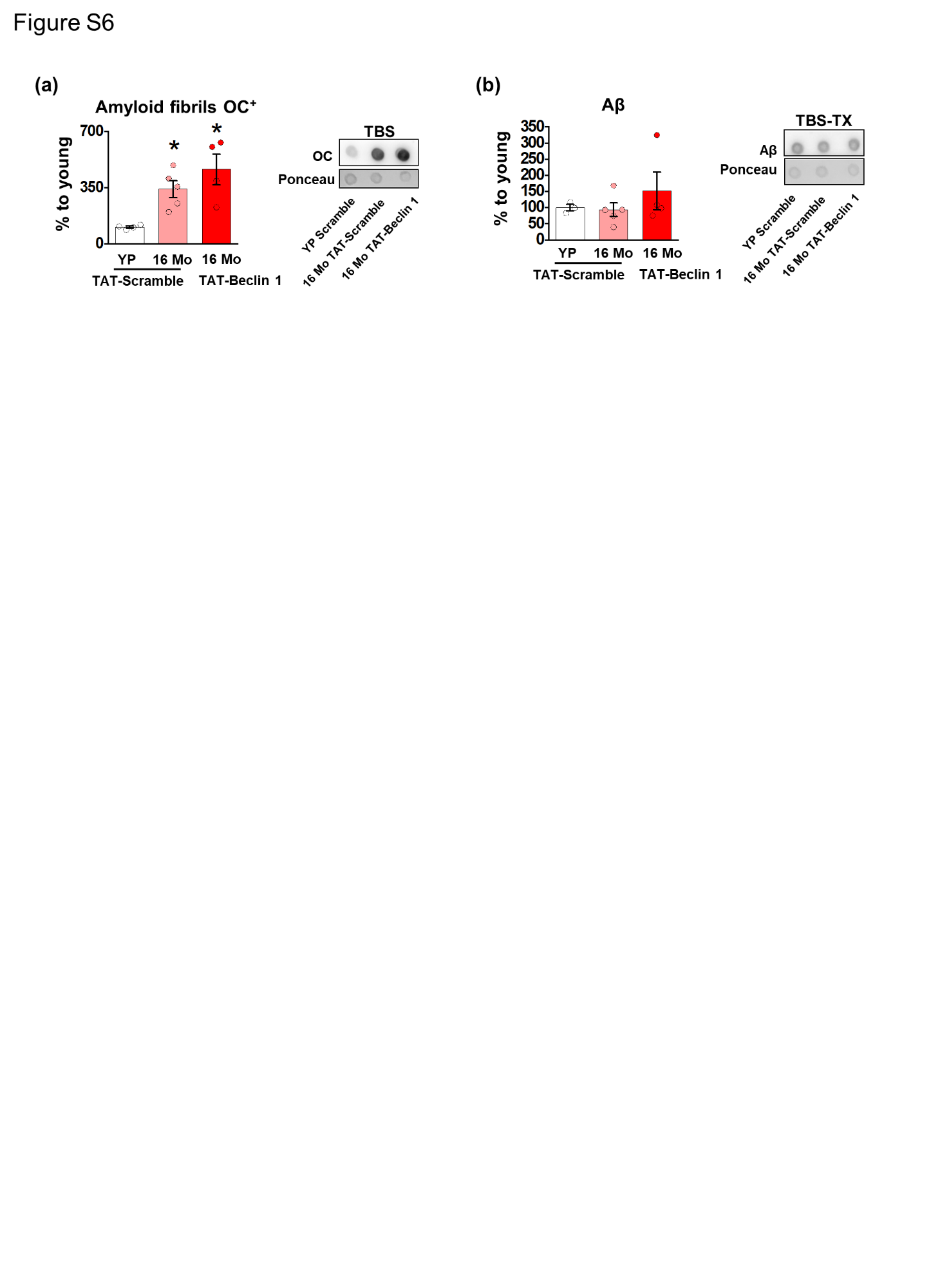
**

**(a-b).** OC^+^ amyloid fibrils levels in TBS extracts and Aβ levels in TBS-TX extracts after chronic treatment of TAT-beclin 1 [OC^+^ amyloid fibrils: F_2,10_=8.16, p=0.007]. Representative dot blots for each condition are presented [YP TAT-Scrambe n≥3; 16 Mo TAT-Scramble n=5; 16 Mo Tat-Beclin 1 n=4]. Bar charts represent mean ± S.E.M.
